# Structural Equation Modelling of Socioeconomic Status on Malnutrition Among Urban Preschoolers: Multi-Group Analysis of Hungary and Indonesia

**DOI:** 10.3390/ijerph23070858

**Published:** 2026-06-30

**Authors:** Arie Dwi Alristina, Éva Kovács, Diyah Arini, Helga Judit Feith

**Affiliations:** 1 Health Sciences Division, Doctoral College, Semmelweis University, 1085 Budapest, Hungary; alristina.arie@phd.semmelweis.hu; 2 Nutrition Department, Sekolah Tinggi Ilmu Kesehatan Hang Tuah Surabaya, Surabaya 60244, Indonesia; 3 Faculty of Health Sciences, Department of Morphology and Physiology, Semmelweis University, 1085 Budapest, Hungary; 4 Nursing Department, Sekolah Tinggi Ilmu Kesehatan Hang Tuah Surabaya, Surabaya 60244, Indonesia; diyaharini76@gmail.com; 5 Department of Social Sciences, Faculty of Health Sciences, Semmelweis University, 1085 Budapest, Hungary; feith.helga@semmelweis.hu

**Keywords:** malnutrition, socioeconomic status, PLS-SEM, pathways analysis, preschoolers

## Abstract

**Highlights:**

**Public health relevance—How does this work relate to a public health issue?**

- It approaches childhood malnutrition as an important social determinant of health and explores the common drivers of preschoolers’ nutritional outcomes across vastly different economic contexts.
- It explores the various and localised pathways linking systemic resource deficits (for example, food insecurity) with non-material factors (such as maternal nutrition knowledge) that link household disadvantage with child health.

**Public health significance—Why is this work of significance to public health?**

- This research demonstrates that the main causes of malnutrition vary widely across contexts. It points out severe resource limitations in developing areas such as Indonesia, while highlighting non-material and behavioural factors in countries with established safety protections, such as Hungary.
- This study recognises that socioeconomic and behavioural models have limited predictive power. It underscores the complexity of childhood malnutrition and stresses the need to consider wider biological and environmental factors in public health evaluations.

**Public health implications—What are the key implications or messages for practitioners, policymakers and/or researchers in public health?**

- In resource-limited settings such as Indonesia, policymakers need to focus on structural reforms to improve family food security, support household incomes, and expand equitable access to basic health care.
- In higher-income countries that have welfare structures in place, such as Hungary, public health practitioners may focus on implementing targeted behavioural change initiatives to reduce maternal stress and optimise the nutritional guidance included in existing social programs.

**Abstract:**

**Background:** Child malnutrition is a major public health problem worldwide, but the pathways through which socioeconomic status (SES) affects nutritional outcomes differ markedly between economic and welfare regimes. Differentiating whether these impacts are direct or through behavioural and structural factors is key to focused policy intervention. **Objective:** This study aimed to investigate the effects of socioeconomic inequalities, maternal knowledge and feeding practices, and food insecurity on preschool undernutrition in Hungary and Indonesia. **Methods:** The study employs a cross-national comparative design. Respondents were households with children 36 to 59 months old; the sample comprised 128 households in Budapest, Hungary and 535 households in Surabaya, Indonesia. The instruments were designed to fit within a survey for data collection. Data were analysed using Partial Least Squares Structural Equation Modelling (PLS-SEM) with Multi-Group Analysis (MGA). **Results:** In Hungary, SES and food insecurity are positively associated with malnutrition, whereas in Indonesia, SES and biological factors drive malnutrition. Intermediate factors such as maternal feeding practices (Hungary) and health coverage (Indonesia) did not directly affect malnutrition. Although the model identified significant socioeconomic pathways, its very low explanatory and predictive power for child malnutrition in both urban Hungary (R^2^ = 0.024) and Indonesia (R^2^ = 0.062), and the predictive relevance (Q^2^ Hungary: −0.009); (Q^2^ Indonesia: 0.016), which remained close to zero or negative, indicate that these variables only partially capture the complex, multifactorial mechanisms driving nutritional status. **Conclusions:** These findings indicate that a more targeted approach to food insecurity and behavioural screening within the Hungarian welfare system would be necessary to address malnutrition effectively. Findings in Indonesia underline the potential benefits of prioritising equity in health coverage and income support.

## 1. Introduction

Malnutrition remains one of the most persistent global public health challenges, affecting a substantial proportion of children under five years of age and exerting lasting consequences upon physical development, cognitive functioning, and economic productivity, most notably within disadvantaged populations. Whilst the proximate determinants of malnutrition, namely inadequate dietary intake [1,2] and the recurrence of infectious disease [3], have been well established in the literature, the underlying structural drivers rooted in socioeconomic [4,5] and wealth-based stratification [6,7] operate through mechanisms that are far more intricate and multi-levelled. These mechanisms vary considerably across different economic systems and welfare regimes, suggesting that the predictive influence of structural determinants is unlikely to be uniform across national contexts. A comprehensive understanding of these dynamics, therefore, requires examination of the interplay between structural determinants and behavioural mediators, including socioeconomic status (SES), household food insecurity, maternal nutritional knowledge, child feeding practices, and eating behaviours.

This study is framed within the United Nations’ 2030 Agenda for Sustainable Development Goals (SDGs). Five goals stand out as particularly important: (1) poverty, (2) hunger, (3) health and well-being, (4) quality education, and (5) reduced inequalities [8]. These aspirations are undergirded by the concept of nutritional equity, ensuring that everyone has equal and just access to adequate and nutritious food [9]. This nutrition equity is key to improving population health, increasing workforce productivity, and reducing growing food inequalities among children [10]. However, despite considerable progress, malnutrition inequalities still represent a significant public health challenge, particularly in low- and middle-income countries (LMICs), where marginalised communities are the most affected [11]. To achieve successful development, policies must prioritise equitable nutrition. This approach is vital for preventing future problems related to impaired growth, cognitive deficits, and decreased work productivity.

Globally, more than 148 million infants and children under five are stunted due to long-term malnutrition, and around 45 million suffer from chronic wasting caused by short-term food deprivation. Sparse 2023 data show a stunting rate of 22.0% among under-fives in LMICs. This data also highlights a rising overweight rate of 5.6% [12], indicating a nutritional transition with economic growth [13]. Structurally, the main drivers of double burden malnutrition are poverty, lower education, and unequal access to healthcare [14]. In high-income countries, established food systems typically provide sufficient, safe, and nutritious food. However, some low-income urban areas, often called food deserts, face significant difficulties in getting affordable fruits, vegetables, and whole grains [15]. The structural pathways by which socioeconomic differences and household environment conditions predict early childhood malnutrition are likely to differ across countries. This underscores the need for comparative studies between high-income and LMICs. This study can evaluate how the influence of these factors changes as economies grow.

Socioeconomic status (SES) is a complex construct that typically includes income, education, occupation, and material assets. At the population level, a lower wealth index or poor economic indicators are linked to widespread undernutrition [16]. As a key driver of nutritional outcomes, SES is crucial, and diets of children from low-SES households are often limited, lacking essential nutrients, which means that these diets fail to support their growth and development. Additionally, maternal education may influence health investment behaviour at various stages of early child development [17] or by altering the risks associated with preterm birth [18]. However, globalisation and market changes have made things more difficult. Ultra-processed foods are now easier to find and sell in low-SES areas [19], making it harder to distinguish between undernutrition and overnutrition [20].

More than 30% of those from low socioeconomic backgrounds in sub-Saharan Africa and South Asia are undernourished [21]. Ethnic minorities in semi-peripheral areas of Eastern Europe, where rapid socioeconomic changes are widening post-socialist divides between rich and poor, often experience high levels (10–30%) of undernutrition [22], in line with minority ethnic groups in South Asia [23]. The continuing burden of child mortality rates continues to highlight gross inequalities in health: deaths attributed to malnutrition remain at levels that are unacceptable by international standards (with the United Nations International Children’s Emergency Fund [UNICEF] citing 12 per 1000 live births as a critical limit) [24], particularly in LMICs such as Indonesia (15 per 1000 live births in 2024) [25]. These levels of death exceed those in many countries that are seen as at risk due to socioeconomic factors [26].

Despite many nutrition programs, child malnutrition continues to be a global issue. These efforts primarily focus on prevalence rates; such an approach overlooks the local factors that influence child health due to socioeconomic disparities [20]. There is increasing awareness that moving policies and programs from one socioeconomic context to another without understanding local causes is not well-supported by evidence and may lead to failure [27]. To create effective interventions, this study needs to go beyond analysing single factors and examine the main and secondary factors that influence nutritional status. Although socioeconomic factors are key drivers, previous studies have typically examined isolated economic indicators, using linear or logistic regression [28]. This method cannot capture the combined effects of food insecurity, maternal nutrition knowledge, child feeding practices, and child eating behaviour.

Our research fills this gap by using Partial Least Squares Structural Equation Modelling (PLS-SEM), a robust multivariate method that estimates multiple interacting pathways within a single model guided by theory [29]. This method is ideal for this study because it allows us to analyse the entire process, from SES to behavioural and knowledge-based factors to child nutrition outcomes, in a single comprehensive analysis. It reveals indirect and mediating relationships that traditional regression misses [30].

The significance of this study lies in its comparative, multi-contextual approach. This study analyses these interconnected pathways in Hungary and Indonesia, which have very different socioeconomic profiles [16]. The present study undertakes a comparative analysis of two markedly contrasting socioeconomic settings: Hungary, a developed European economy with generally well-functioning social welfare systems, and Indonesia, a rapidly developing economy currently confronting the double burden of malnutrition. Although there are major differences in their economies and cultures, the key factors examined, such as household food insecurity and child feeding behaviours, are important for child health worldwide. Recent global frameworks and trusted cross-cultural measures show that these factors hold similar meanings across diverse groups [31]. In Hungary, although social protection systems buffer households against severe poverty, persistent nutritional inequalities continue to affect particular subgroups of the population [32,33]. In contrast, health inequities and high rates of income inequality are still the primary causes of chronic child undernutrition in Indonesia [34]. Utilising these universally acknowledged constructs in such different contexts [35] enables us to distinguish context-specific factors influencing malnutrition from those that are universal. Consequently, the objective of this study is to determine whether socioeconomic inequalities in preschool undernutrition operate through direct pathways or whether they are mediated by behavioural and structural pathways. Ultimately, understanding these dynamics across contexts provides the evidence needed to design interventions to mitigate malnutrition in children.

### Conceptual Framework

**Figure 1:**
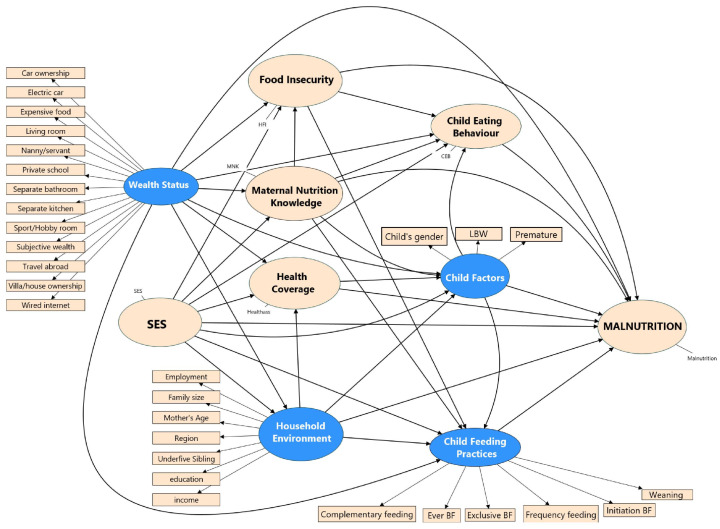
Modified conceptual framework of socioeconomic factors and malnutrition.

This study draws on established nutritional principles to develop a clear framework, modified from UNICEF [36,37], for comparing the causes of child malnutrition in Hungary and Indonesia. Figure 1 shows this condition operating across a continuum of causation, categorising variables into basic, underlying, and immediate factors hypothesised to directly and indirectly influence a child’s nutritional status. This results in a multi-contextual structural equation model (SEM) that examines a range of causes. It groups variables into basic, underlying, and immediate factors that are expected to directly and indirectly influence a child’s nutritional status. At the foundational level, basic or distant causes include broader societal and household factors such as wealth status, SES, and household environment [38]. These fundamental socioeconomic differences influence the quality and availability of resources for the family, which, in turn, affects the underlying or proximate determinants. The underlying factors operate within the household and include food insecurity, maternal nutrition knowledge, health coverage, and child-related factors (such as gender, low birth weight, and prematurity) [39]. Two issues significantly affect the immediate causes of malnutrition, as defined by this model: child eating behaviour and child feeding practices (such as exclusive breastfeeding, initiation of breastfeeding, weaning practices, meal frequency, and complementary feeding) [40,41,42,43]. Overall, this theoretical framework posits how these interconnected pathways may work together to predict child undernutrition. Because of the lack of comparative SEM literature on this specific cross-country comparison, this study formulates an exploratory theoretical proposition. Given the extreme differences in both welfare protection and income equality, this study assumes that structural pathways will remain differentially predictive across country contexts, with a greater impact in Indonesia than in Hungary.

## 2. Methods

### 2.1. Study Design

A comparative cross-sectional study was undertaken in two contrasting urban settings: Surabaya (Indonesia), the capital of East Java province and a major industrial-port city representative of Indonesia’s rapidly urbanising areas, and Budapest (Hungary), a high-income European capital characterised by an established social welfare system. A survey was conducted in Surabaya from May 2024 to April 2025 and in Budapest from January to June 2025, enabling the examination of socioeconomic disparities in child nutrition within a comparable temporal framework. The design followed community health survey guidance to capture the prevalence and predictors of undernutrition among households with children aged 36–59 months.

### 2.2. Data Collection

Survey locations were chosen purposively: ten administrative districts (two from each of the city’s five regions) across Surabaya, where mothers were recruited via Posyandu community health posts, and five kindergartens (two in Pest and three in Buda) in Budapest. In particular, the eligibility criteria included being a biological mother (living in the selected households) of at least one child aged 36–59 months. Children were excluded if diagnosed with chronic illness (e.g., asthma, congenital heart disease, or chronic allergies), severe acute illness (e.g., upper respiratory infections, fever, and diarrhoea), or significant deformity of the spine or lower extremities; furthermore, in households with multiple eligible children, only one was included, chosen by random selection. The minimum sample size was estimated using GPower 3.1.9.4, with α = 0.05, power (1 − β) = 0.95, a medium effect size (f^2^ = 0.20), and nine latent predictors, yielding 127 households. Of the 657 households initially screened in Surabaya, 535 met this age criterion, and of the 132 approached households in Budapest, complete data were available for 128. The weights and heights of children were measured using a portable SECA 813 electronic flat scale and the Shorr board. The questionnaire was pretested with 10 participants, a sample size considered sufficient for pilot testing [44]; equipment was calibrated using standard weights, and trained enumerators were taught how to deliver questions, record responses, and participate in simple role-play exercises to better understand the study’s flow. The instrument was translated from English to Bahasa Indonesia (certified by Muhammadiyah Surabaya University) and Hungarian (certified by Semmelweis University).

### 2.3. Variables and Data Sources

Data were collected via standardised, culturally adapted questionnaires and anthropometric measurements. Data were gathered through structured interviews using a five-part questionnaire translated into Hungarian and Indonesian and certified accordingly.

#### 2.3.1. Anthropometric Measurements

The dependent variable in this study refers to child nutritional status, which was defined as the Z-score of weight-for-age (WAZ), height-for-age (HAZ), and weight-for-height (WHZ), calculated using WHO ANTHRO 3.2.2 software, which is validated by the World Health Organisation (WHO). Z-scores were calculated for WAZ, HAZ, and WHZ below −2 SD, indicating stunting, wasting, or underweight, respectively. To clarify the coding direction for the analysis, the malnutrition dependent variable was coded as a binary outcome. A value of 1 indicates better nutritional status (Z-score ≥ −2; not undernourished), and a value of 0 indicates undernutrition.

#### 2.3.2. Socioeconomic Status (SES)

SES was conceived as a formative latent construct containing household income (quintile-transformed), maternal education (UNESCO-coded ordinal scale), employment status (binary variable), subjective wealth perception (Likert scale), and, lastly, asset ownership, including car and house/villa (binary variables). We determined composite scores by combining all indicators within each domain, with any missing data scored as 0 and the maximum score being equal to the number of items in that respective domain, then categorised the continuous composite scores into tertiles (≤33.33%, 33.34–66.67%, and >66.67%), which was done to divide the sample into three equal, relative socioeconomic strata (low, middle, and high), respectively [45].

#### 2.3.3. Household Food Insecurity

Food insecurity was assessed using the Food Insecurity Experience Scale Survey Module (FIES-SM), a validated instrument consisting of 8 yes/no items that inquire whether respondents experienced various outcomes over the past 12 months. Total scores were based on the raw scores (0–8): food-secure households had a score of 0, and all others were classified as food-insecure (score ≥ 1).

#### 2.3.4. Maternal Nutrition Knowledge

Maternal nutrition knowledge was assessed using a 16-item, translated version of the General Nutrition Knowledge Questionnaire–Revised (GNKQ-R), dietary recommendations, nutrient sources, and healthy eating principles. Each correct response scored 1, and each incorrect response scored 0. Nutrition knowledge was assessed using a modified General Nutrition Knowledge Questionnaire-Revised (GNKQ-R), which contains 16 core questions. Because several of these are matrix-style questions having multiple sub-items that will be scored separately (i.e., classifying multiple distinct food items in one question), the maximum cumulative score is 52. Total scores were divided into three levels of knowledge: low (≤17), moderate (18–34), and high (≥35).

#### 2.3.5. Child Feeding Practices and Eating Behaviour

Attainment of Infant and Young Child Feeding (IYCF) practices was evaluated against WHO/UNICEF of IYCF indicators, including initiation of breastfeeding within an hour, exclusive breastfeeding at different times, prompt weaning practices, timely introduction of complementary foods, and age-appropriate meal frequency for infants. Child eating behaviour was assessed using the 35-item Child Eating Behaviour Questionnaire (CEBQ), with eight subscales from both the food approach and food avoidance domains measured on a five-point Likert scale. Latent Profile Analysis (LPA) was conducted in RStudio 4.5.2 to delineate unique profiles of use-related behaviours.

#### 2.3.6. Child Factors

The characteristics of children (child-related factors) were included as formative indicators in the structural model as follows: (1) the child’s gender, expressed as the percentages of boys and girls in this survey; (2) low birth weight for the child, ≤2500 g at birth; and (3) preterm birth or premature birth, for infants born before 37 completed weeks of gestation [46].

#### 2.3.7. Wealth Status and Household Environment

This study defined Wealth Status as a formative latent variable within the SES framework. The potential indicators for households included owning a house or villa, having a car or electric car, spending on expensive food, having a living room, employing a paid nanny or servant, affordability of private school, having separate bathroom and kitchen facilities, and engaging in arts or sports, along with wealth being assessed through subjective measures such as travel abroad and access to the internet. Although the household environment is not directly observed, this is an important latent factor in the observed variables. Indicators of household environment include maternal education attainment, parent employment status, family size, income level, mother’s age, the number of siblings under five, and region. This environment creates a household context that helps explain the differences in nutrition among populations. SES and Wealth Status were considered conceptually similar, as they shared common indicators, but empirical tests confirmed them as functionally distinct constructs in this model, enabling an integrated analysis of their pathways. Furthermore, evidence for discriminant validity, assessed using the Heterotrait–Monotrait (HTMT) ratio, indicated that SES and Wealth Status operated as empirically distinct constructs in the model despite conceptual overlap, permitting investigation of their subtle independent pathways.

### 2.4. Data Analysis

Preliminary processing of data was performed by SPSS 27.0 and R 4.5.2, while PLS-SEM was executed using SmartPLS 4 software. The choice of PLS-SEM was justified because it is appropriate for managing complex formative and predictive constructs. The measurement model was examined by composite reliability (CR) with a value ≥ 0.60 and average variance extracted (AVE) with a value ≥ 0.50; the structural model fit was assessed using Standardised Root Mean Square Residual (SRMR), with the model defined as a good fit if the value < 0.10. Variance Inflation Factors (VIF) were used to assess multicollinearity. We estimated mediation effects using the product-of-coefficients approach with bootstrapped confidence intervals. To compare path coefficients and predictive relevance (Q^2^) between the samples of Hungary and Indonesia, this study used Multi-Group Analysis (MGA) in SmartPLS4. This study reported direct, indirect, and total effects as standardised coefficients and odds ratios; statistical significance was defined as *p* < 0.05. Listwise deletion was employed for missing data with a value below 5%.

The data analysis proceeded in four stages:

1. Measurement Model Assessment: Evaluating indicator reliability, internal consistency CR (Cronbach’s Alpha), convergent validity: AVE (Average Variance Extracted), and discriminant validity using HTMT Ratio of correlations.
2. Structural Model Assessment: Assessing collinearity with VIF, path coefficients, statistical significance (via 5000 bootstrap resamples), coefficient of determination (SRMR), and predictive relevance (Q^2^).
3. Measurement Invariance of Composite Models (MICOM): Group comparison in PLS-SEM will be misleading without the establishment of the invariance of construct measurement. This was required because the differences in these structural relationships could have represented either socioeconomic differences between Hungary and Indonesia or incongruence in the structural and behavioural contexts of these survey items.
4. MGA: This was conducted to test for significant differences in the path coefficients between the Hungarian and Indonesian cohorts.

### 2.5. Ethical Considerations

Ethics Approval/Consent for Participation was obtained from the Health Research Ethics Committee—National Institute of Health Research and Development, Indonesia; 039/KE.03/SK/02/2024. Additionally, Hungary’s Regional ethical approval was obtained from Regionális és Intézményi Tudományos Kutatásetikai Bizottság, Hungary; SE RKEB 207/2024. Written informed consent was obtained from all participants before enrolment; thumbprints witnessed by a third party were accepted for illiterate participants. All responses were anonymous, and participation was voluntary.

## 3. Results

### 3.1. Population Sociodemographic Characteristics

The descriptive analysis demonstrates a marked distinction between the two study populations. Malnutrition occurs in only 11.7% (*n* = 15) of Hungarian children, but in 50.1% (*n* = 268) of Indonesian children. The majority of the sample is from a middle-SES group in Hungary, and malnutrition was found in 27.8% of that group, as well as in the high-SES group, at 9.1%. Similarly, in Indonesia, among the subgroup of children with low SES, 70.4% were malnourished, compared to only 27.6% of those with high SES. Household food insecurity also played a higher role in Indonesia. In that country, 55.4% of food-insecure households had malnourished children, while the rate was 46.8% for food-secure households. Maternal factors showed distinct trends. In Hungary, mothers with a moderate education level tended to have a 14.1% chance of having malnourished children. In Indonesia, low maternal educational attainment affected 60.0% of the children experiencing malnourishment. Regarding biological and feeding factors, both countries exhibited significant associations with malnutrition. In Hungary, 50.0% of low birth weight children were malnourished, while the figure was 62.0% in Indonesia. In Hungary, preterm birth children tend to have a risk of being malnourished of 33.3%, while in Indonesia, the equivalent measurement is 53.3% (Table A1).

In addition, Child Feeding Practices in Hungary showed a lower percentage of malnourished children than in Indonesia. Inappropriate age at the start of complementary feeding in Hungary, specifically, less than 6 months and more than 6 months, was at 10.8% and 14.3% of malnourished children, respectively. Whereas in Indonesia, the figures were 53.2% and 53.2%, respectively. In Hungary, breastfeeding initiation occurred within 1 h postpartum in the majority of cases (88.8% of well-nourished), along with similar determinations for ever breastfeeding (88.1% of well-nourished) and prevalent exclusive breastfeeding (88.3% of well-nourished). These practices reflected the established healthcare protocols and maternal knowledge in a high-income setting. In Indonesia, initiation of breastfeeding was less consistently early (53.5% were initiated more than 1 h after birth and became malnourished); though ever breastfeeding remained prevalent, exclusive breastfeeding was less universally practised (51.7% children without exclusive breastfeeding being malnourished) than in Hungary (11.8%), reflecting diverse infant feeding practices influenced by maternal knowledge, employment patterns, and cultural factors in the Indonesian context (Table A1).

### 3.2. Prevalence of Malnutrition and Socioeconomic Status

**Figure 2:**
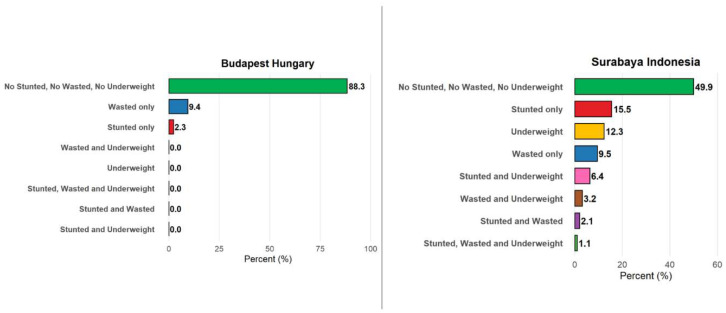
Malnutrition distribution (*n* = 128, Hungary; *n* = 535, Indonesia) (Source: Primary Data Survey).

The prevalence of undernutrition among children under five years varied substantially between the two settings. Malnutrition prevalence, operationalised through anthropometric indicators (stunting, wasting, and underweight status), differed substantially between Hungary and Indonesia. In Hungary, 88.3% of children were classified as non-malnourished, based on composite anthropometric criteria, compared to 49.9% of children in Indonesia (Figure 2).

**Figure 3:**
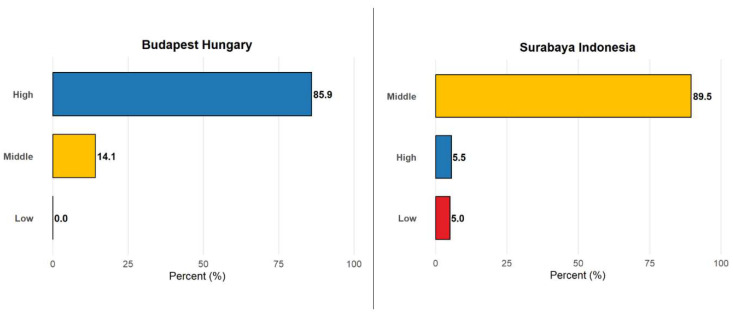
Socioeconomic status distribution (*n* = 128, Hungary; *n* = 535, Indonesia) (Source: Primary Data Survey).

As shown in Figure 3, the SES distribution varied significantly by country. In the Hungarian sample, the households were characterised primarily by high-SES households (85.9%), as indicated by income, employment status, educational attainment, and subjective perceptions of wealth. The second portion was in the middle-SES group (14.1%), with virtually no low-SES households represented (0%). This distribution is indicative of an ‘overgrown’ high-income European nation with income and living standards that are roughly equalised. In contrast, in Indonesia, SES was less homogeneous; middle-SES households dominated (89.5%), followed by high-SES households (5.5%) and low-SES households (5.0%). Such a tripartite distribution is representative of the economic polarisation typical of rapidly urbanising LMICs.

### 3.3. Chi-Square Associations with Latent Variables

**Figure 4:**
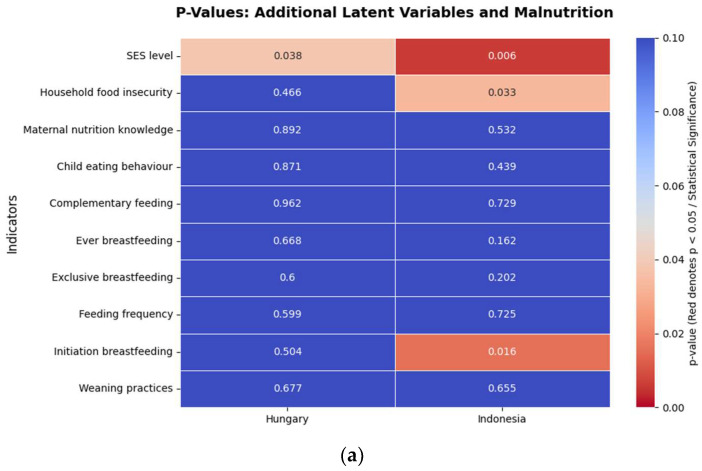

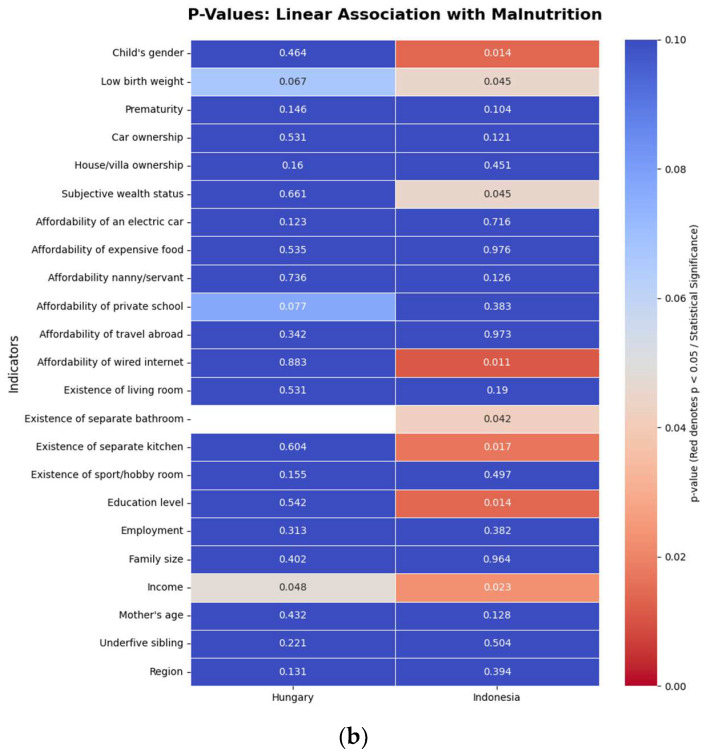
Heatmap of chi-square associations between malnutrition, latent variables, and indicators; (**a**) bivariate analysis of latent variables (*n* = 128, Hungary); (**b**) bivariate analysis of construct variables (*n* = 535, Indonesia), (Source: Primary Data Survey).

Figure 4 shows the chi-square relationships between malnutrition and all latent variables and their indicators. In Hungary, significant relationships were found only in the socioeconomic area. These involved the latent concept of SES (χ^2^, *p* = 0.038) and the household income (χ^2^, *p* = 0.048) indicator within the household environment category. In Indonesia, relationships extended across several latent areas. This indicated that malnutrition was linked to SES (χ^2^, *p* = 0.006); household food insecurity (χ^2^, *p* = 0.033); child-related factors such as gender (χ^2^, *p* = 0.014) and low birth weight (χ^2^, *p* = 0.045); wealth indicators, including subjective wealth perception (χ^2^, *p* = 0.045) and material assets; household environment elements such as maternal education (χ^2^, *p* = 0.014) and income (χ^2^, *p* = 0.023); and child feeding practices, especially the timing of early breastfeeding initiation (χ^2^, *p* = 0.016). This pattern confirms the complicated and multi-level causes of malnutrition in LMICs.

### 3.4. Measurement Model Assessment: Outer Model Validity and Reliability

The measurement model was measured using three components in the study, namely, collinearity assessment, convergent validity, and discriminant validity, to assess latent constructs and their reflective and formative indicators.

#### 3.4.1. Collinearity Assessment and Indicator Validity

Table 1 presents the VIF analysis for formative constructs. For Hungary, all indicator variables had VIF values below 3. This shows that the construct specification is valid and there are no issues with collinearity. Similarly, the VIF in Indonesia remains below 3 for all indicators. This confirms the absence of multicollinearity and ensures that each formative indicator provided independent, unique information to its respective construct. These findings in both settings support the stability of estimated path coefficients in the structural equation model. Each construct includes non-redundant dimensions. The difference in VIF values between the Hungarian and Indonesian samples, while within an acceptable range, suggests that SES may correlate differently in wealthier and emerging-economy contexts.

#### 3.4.2. Discriminant Validity Through HTMT Ratios

Discriminant validity ensures that distinct latent constructs measure different phenomena. This study assesses the HTMT ratios. Table 2 displays the HTMT between SES and wealth status, treating human capital as distinct from humans’ material capital (as in Table 2). The HTMT ratio of 0.445 in Hungary was far below the threshold of 0.85, thus confirming discriminant validity. The separation between constructs was even clearer, as indicated by the HTMT ratio of 0.025 in Indonesia (Table 2). However, this implies that the wealth dimension of SES operates more independently in this geographical context. This might indicate different pathways wherein structural factors (education and employment), versus resources accumulated across the life span (housing and assets), affect child nutrition.

#### 3.4.3. Convergent Validity Through Outer Loadings

The outer loadings of latent variables and indicators are shown in Table A2 after removal of the underperforming constructs. In Hungary, only low birth weight (0.779) and prematurity (0.873) remained valid indicators for child factors. Child gender was excluded due to low loading. As for affordability indicators reflecting wealth status, electric car (0.762), expensive food (0.684), nanny services (0.776), private school (0.714), and travel abroad (0.654) were retained. Material assets, such as house and car ownership, were removed from the Hungarian model due to low loadings. Education level (0.564) and family income were retained, while employment, family size, and region variables were excluded. For child feeding practices, complementary feeding timing (0.695), ever breastfeeding (0.658), exclusive breastfeeding (0.830), and breastfeeding initiation (0.565) were retained. However, both feeding frequency and weaning practices were excluded as predictors of IYCF practices, even though they remain important aspects of child feeding practices.

In Indonesia, child factors included gender (0.768) and low birth weight (0.602), while prematurity (0.305) was well below the acceptable threshold. Wealth status indicators were retained, including car ownership (0.571), affordability of an electric car (0.637), the ability to afford expensive food (0.691), having a nanny/servant (0.694), and affordability of private school (0.734). The household environment included education (0.772) and income (0.857), both of which remained acceptable. Employment and region were removed from the Indonesian model. Child feeding practices included ever breastfeeding (0.176, removed), exclusive breastfeeding (0.676, retained), and feeding frequency (0.517, retained). However, complementary feeding (0.540) and weaning practices (0.534) were retained.

**Figure 5:**
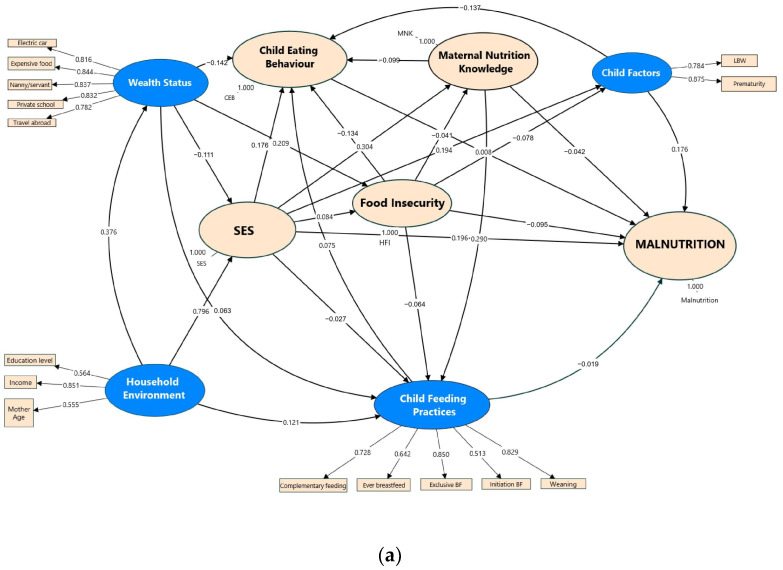

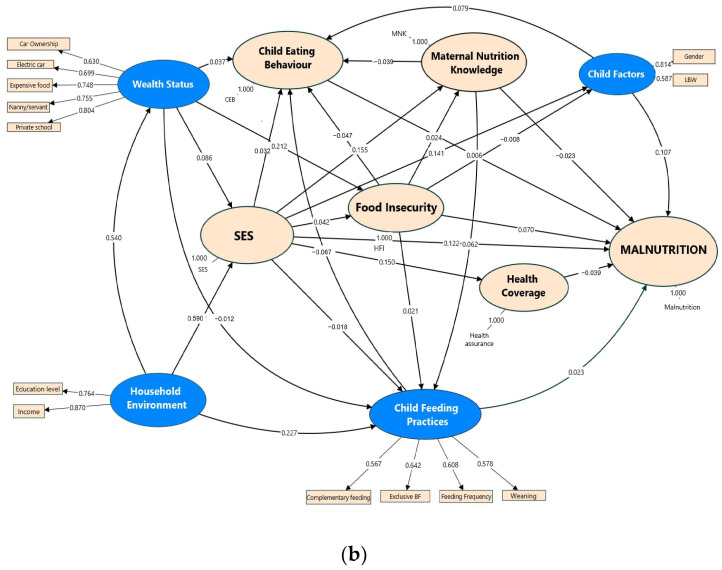
Path diagrams of socioeconomic factors relative to malnutrition, (**a**) Hungarian model (*n* = 128); (**b**) Indonesian model (*n* = 537) (Source: Primary Data Survey, PLS-SEM Test).

Figure 5 illustrates the refined measurement models for Hungary and Indonesia, respectively, highlighting all retained indicators and their loadings after removing marginal outer loading. It shows that all indicators remain acceptable in both models.

#### 3.4.4. Composite Reliability and Average Variance Extracted

CR and AVE were performed for all latent constructs. In Hungary, all constructs achieved composite reliability exceeding 0.60 (threshold for adequate reliability), and AVE exceeding 0.50 (threshold for convergent validity). Specifically, the Hungarian model demonstrated CR for child factors of 0.816 and √AVE of 0.831; wealth status CR of 0.913 and √AVE of 0.822; household environment, CR of 0.702 and √AVE of 0.671; and child feeding practices, CR of 0.842 and √AVE of 0.723. The Indonesian model showed child factors CR of 0.665 and √AVE 0.710; wealth status, CR of 0.851 and √AVE of 0.598; household environment, CR of 0.802 and √AVE of 0.819; and child feeding practices, CR of 0.690 and √AVE of 0.701, confirming that the measurement instruments are consistently reliable and valid, capturing underlying constructs in both national contexts. These metrics substantiate proceeding to structural model analysis, as shown in Table A3 and Table A4.

### 3.5. Inner Model Fit

#### 3.5.1. Explained Variance and Model Predictive Power

The inner model fit, assessing relationships between latent variables, was evaluated through R^2^ (coefficient of determination) values indicating explained variance in endogenous variables, shown in Table A5. R^2^ analysis demonstrated that, while the structural model offered moderate predictive value for SES in both countries, it was weak in predicting child malnutrition in both countries. Setting Hungary aside (R^2^ = 0.062), the Indonesian model results indicate a lower predictive power (R^2^ = 0.024).

#### 3.5.2. Model Fit Indices

The SRMRs for the Hungarian model (0.090) and for the Indonesian model (0.078) are both lower than 0.08, which is an acceptable fit, as presented in Table 3. Predictive relevance (Q^2^) showed that each construct has a different ability to predict the outcome. SES revealed a strong predictive relevance in Hungary (Q^2^ = 0.564) and wealth status (Q^2^ = 0.283). The variables with less predictive relevance were child eating behaviour and malnutrition, which reached a Q^2^ < 0. In Indonesia, the weak predictive relevance with Q^2^ does not exceed 0.15 for malnutrition (Q^2^ = 0.016) and child factors (Q^2^ = 0.017). Maternal nutrition knowledge (Q^2^ = 0.038), household food insecurity (Q^2^ = 0.044) and child feeding practices (Q^2^ = 0.024) also have moderate predictive relevance on other latent variables. The predictive relevance (Q^2^ = 0.283) for this model showed that wealth status is highly predictive. Both country models have weak predictive power for malnutrition. Consequently, while many significant individual pathways have emerged in the models for both countries, the overall structural models do not robustly predict malnutrition, further confirming the complex and multifaceted nature of this outcome.

### 3.6. Direct Effects in the Structural Model

#### 3.6.1. Hungarian Direct Effects Pathways

Table A6 presents the direct effect results for the Hungarian structural model, indicating that several pathways were statistically significant at *p* < 0.05. Child factors had a significant negative effect on child eating behaviour (β = −0.137, *p* = 0.002), while food insecurity significantly predicted child factors (β = −0.078, *p* = 0.003) and malnutrition (β = −0.095, *p* = 0.001). Household environment showed significant positive effects on SES (β = 0.796, *p* < 0.001) and wealth status (β = 0.376, *p* < 0.001). Maternal nutrition knowledge had a significant positive effect on child feeding practices (β = 0.290, *p* < 0.001), and SES significantly predicted child eating behaviour (β = 0.171, *p* = 0.004), malnutrition (β = 0.196, *p* = 0.027), and maternal nutrition knowledge (β = 0.304, *p* < 0.001). In addition, wealth status had significant effects on child eating behaviour (β = −0.142, *p* = 0.023), Food insecurity (β = 0.209, *p* < 0.001), and SES (β = −0.111, *p* = 0.030). In contrast, the remaining direct pathways, including the effects of child eating behaviour, child feeding practices, maternal nutrition knowledge, child factors, and several SES-related pathways on malnutrition or behavioural outcomes, were not statistically significant. Overall, these findings suggest that in the Hungarian sample, malnutrition was directly predicted by food insecurity and SES, while other determinants mainly influenced intermediate constructs such as child eating behaviour, child feeding practices, maternal nutrition knowledge, and socioeconomic conditions.

#### 3.6.2. Indonesian Direct Effects Pathways

Table A7 presents the direct effect results for the Indonesian structural model, indicating that several pathways were statistically significant (*p* < 0.05). Child factors had a significant positive effect on malnutrition (β = 0.107, *p* = 0.021), while household environment significantly predicted child feeding practices (β = 0.227, *p* = 0.002), SES (β = 0.596, *p* < 0.001), and wealth status (β = 0.540, *p* < 0.001). SES showed significant positive direct effects on child factors (β = 0.141, *p* = 0.001), health coverage (β = 0.150, *p* < 0.001), malnutrition (β = 0.122, *p* = 0.003), and maternal nutrition knowledge (β = 0.155, *p* < 0.001). In addition, wealth status had significant positive effects on food insecurity (β = 0.212, *p* < 0.001) and SES (β = 0.086, *p* = 0.035). These findings suggest that in the Indonesian sample, child factors and SES directly predicted malnutrition. Meanwhile, household environment, wealth status, and SES mainly influenced intermediate factors linked to socioeconomic conditions, health coverage, maternal nutrition knowledge, food insecurity, and child feeding practices.

### 3.7. Indirect Effects and Mediation Pathways

#### 3.7.1. Mediation Analysis for Hungarian Model

**Figure 6:**
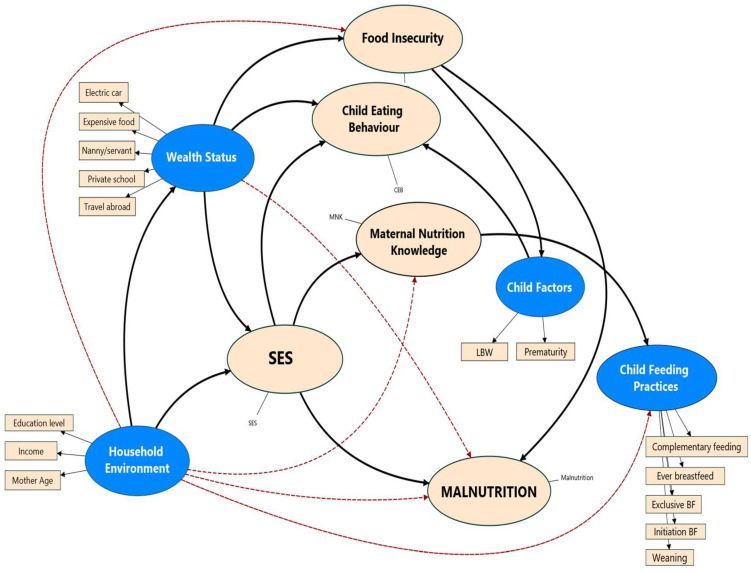
Model path diagram of socioeconomic disparities on nutrition inequalities in Hungary (*n* = 128, Hungary; Source: Primary Data Survey, PLS-SEM test).

The results of the direct-effect analysis for the Hungarian structural model are shown in Table 4 and Figure 6. The PLS-SEM path analysis reveals that only two latent variables exert statistically significant direct influences on child malnutrition. SES demonstrated a significant positive direct effect on malnutrition (*p* < 0.05), because the dependent variable was coded such that higher values indicate (better nutritional status/higher anthropometric score = 1); this positive association suggests that a higher SES leads to better nutrition results. Meanwhile, household food insecurity showed a highly significant direct effect (*p* < 0.01). These findings indicate that SES and food insecurity remain direct predictors of malnutrition. In contrast, the direct pathways for child factors, child eating behaviour, child feeding practices, maternal nutrition knowledge, and household environment did not meet statistical significance on child malnutrition. This indicates that the latent variables included in the model do not directly influence malnutrition among preschool children in Hungary, but they may contribute to lower predictive relevance in the Hungarian sample.

The mediation path analysis for the Hungarian sample identified several significant indirect pathways that influence child malnutrition, as described in Table 5. The household environment had a significant indirect effect on child malnutrition through SES as a mediator (β = 0.156, *p* = 0.030). The similar path from household environment through SES was also associated with maternal nutrition knowledge (β = 0.242, *p* = 0.000), indicating that SES was an important mediator linking maternal knowledge, which may improve maternal awareness of child feeding practices and child eating behaviour. Furthermore, this finding confirmed that the household environment influenced child eating behaviour (β = −0.053, *p* = 0.036) and household food insecurity (β = 0.078, *p* = 0.000) through wealth status. Wealth status showed significant indirect effects on child factors (β = −0.016, *p* = 0.017) and on child malnutrition (β = −0.020, *p* = 0.010) through food insecurity. Additionally, a pathway from the household environment to malnutrition, mediated by wealth status and food insecurity, was confirmed to be significant (β = −0.007, *p* = 0.014).

#### 3.7.2. Mediation Analysis for Indonesian Model

The mediation analysis of direct effect pathways in the Indonesian structural model showed a very limited ability to predict child malnutrition. As shown in Table 6, among the model variables, SES and child factors had statistically significant direct effects on child malnutrition outcomes. SES and child factors remained the key factors significantly affecting child malnutrition, with SES partially mediating (both direct and indirect effects), whereas child factors showed no mediation. Correspondingly, other variables, such as household food insecurity, maternal nutrition knowledge, child eating behaviour, child feeding practices, health coverage, household environment, and wealth status, did not reveal significant direct pathways to child malnutrition. This pattern suggests that, in Indonesia, the structural factors commonly associated with malnutrition have less direct explanatory power.

**Figure 7:**
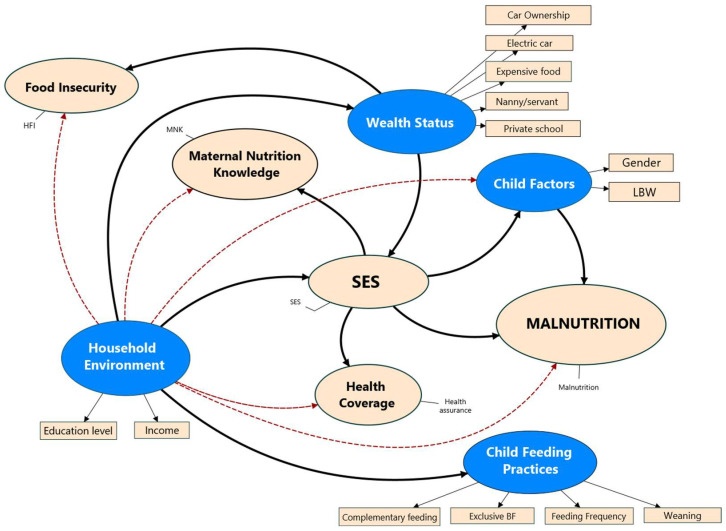
Model path diagram of socioeconomic disparities on nutrition inequalities in Indonesia (*n* = 535; Source: Primary Data Survey, PLS-SEM test).

The mediation path analysis for the Indonesian model is presented in Table 7 and Figure 7 and shows several indirect pathways linking child malnutrition and its related latent variables. A significant pathway from household environment to SES, and then to child factors, and finally to malnutrition, was observed (β = 0.009, *p* = 0.049). This study suggests that SES played a mediating role in translating household environment into child nutrition outcomes. The household environment had significant indirect effects on several latent variables as well, including child factors (β = 0.084, *p* = 0.002), malnutrition (β = 0.073, *p* = 0.006), health coverage (β = 0.089, *p* = 0.000), and maternal nutrition knowledge (β = 0.092, *p* = 0.000); these variables were mediated by SES. Additionally, the household environment significantly indirectly affected household food insecurity (β = 0.114, *p* = 0.000) through wealth status as a mediating variable.

### 3.8. Measurement Invariance Assessment: MICOM Procedure

The SEM-MGA requires establishing measurement invariance to ensure that the latent constructs are measured equivalently across groups. The MICOM three-step procedure examined configural, compositional and scalar invariance. Utilising similar latent variables with retained indicators in both models, namely, the Hungarian model and Indonesian model, and by equivalent indicator–construct relationships, Step 1 confirmed a configural invariance. This step affirms that both samples share the same constructs and measurement structures [47].

This study was assessed by comparing original composite correlations between countries to reference distributions of the same size from among 5000 permuted composites (Step 2—Compositional Invariance). In comparing Hungary and Indonesia, all composite score correlations were above the 95% percentile of the permutation distribution, demonstrating compositional invariance and justifying the determination that composite scores measure essentially equivalent constructs in different countries [48]. This finding provides support for interpreting differences in structural coefficients as evidence of country differences rather than measurement artefacts.

The result of MICOM Step 2 in Table 8 assessed compositional invariance between the Hungarian and Indonesian models. It showed that compositional invariance was established for child eating behaviour, food insecurity, malnutrition, and maternal nutrition knowledge. The original correlation values were equal to 1 and exceeded the 5.00% permutation quantile, with permutation *p*-values above 0.05. These findings suggest that the composite scores for these groups were derived equally in both countries, allowing significant comparisons in MGA. While adequate measurement equivalence was established for most constructs, indicating that the SEM model may be justifiably tested across countries, interpretations involving SES should be made cautiously, as its composite structure is not entirely equivalent across groups. Therefore, this study revealed that compositional invariance was not established for SES with a permutation *p*-value < 0.05 (*p* = 0.028), indicating that SES was represented differently between the Hungarian and Indonesian samples.

Step 2 of the MICOM procedure tested for compositional invariance (Table 8). The results of the analyses show that child eating behaviour, food insecurity, malnutrition, and maternal nutrition knowledge were identified as compositional-invariant; permutation *p*-values exceeded 0.05 for these areas. Compositional invariance was, however, not found for socioeconomic status (SES) (*p* = 0.028). This confirms that SES is more or less invariant and, hence, quantitatively supports the idea inherent in the conceptualisation of SES as a variable with very different implications for, and significant relationships to, socio-economic reality across the cultural dimensions between Hungary and Indonesia.

MICOM Step 3 results in Table 9, which assesses the equality of mean values and variances between the samples from Hungary and Indonesia. The results demonstrated a significant permutation *p*-value for child eating behaviour, food insecurity and malnutrition (*p* = 0.000); hence, no inequality was established. This means that these factors differed significantly between the two country groups, suggesting contrasts in child eating behaviour, household food insecurity, and malnutrition. Conversely, maternal nutrition knowledge and SES were determined to be equal, as their permutation *p*-values are above 0.05 (*p* = 0.092 and *p* = 0.370, respectively). These differences between Hungary and Indonesia for these factors were not significant at the 95% confidence level. These findings indicate that full measurement invariance across constructs could not be established. Maternal nutrition knowledge and SES indicated equality, whereas child eating behaviour, food insecurity, and malnutrition showed significant differences.

In order to perform Step 3 (shown in Table 9), the equality of composite mean values and variances was evaluated. Partial measurement invariance is confirmed for the model because compositional invariance was established for four of the five constructs. Therefore, this study continued with the MGA to compare the structural models of both countries. Yet, the cross-country comparisons of path coefficients that include SES in Step 2 remain exploratory and should be interpreted with caution, as compositional invariance for SES was not established.

### 3.9. Multi-Group Analysis: Comparing Structural Relationships Between Hungary and Indonesia

Table 10 presents the MGA results, highlighting parametric significance tests comparing path coefficients between Hungary and Indonesia. Overall, there were statistically significant differences between the two countries (*p* < 0.05) in three critical pathways: SES to food insecurity, SES to malnutrition, and SES to maternal nutrition knowledge. This test confirms the validity of the comparative study across the Indonesian and Hungarian samples.

Findings from the MGA for Hungary and Indonesia showed that most structural paths were not significantly different, indicating that the core mechanisms driving child malnutrition are similar in both contexts. However, significant differences were identified in three specific pathways: the effects of SES on household food insecurity, malnutrition, and maternal nutrition knowledge (*p*-values < 0.05). These findings suggest that SES has a highly context-specific effect rather than representing entirely different theoretical mechanisms across countries. Ultimately, the commonality in model structure is that both populations shared a similar core regarding food security, but differential associations were observed for SES on direct nutritional outcomes, maternal nutrition knowledge, and food insecurity. These findings underscore the need for locally tailored, SES-sensitive policy interventions.

## 4. Discussion

### 4.1. Cross-Country Interpretation Using the Modified Framework

This study used a modified version of UNICEF’s framework to identify basic, underlying, and immediate causes of child undernutrition in urban preschoolers in Hungary and Indonesia. For Hungary, the model showed smaller socioeconomic differences in malnutrition. SES and wealth status exerted effects that were more subtle and partially indirect. In contrast, findings from Indonesia highlighted SES as a key factor in nutritional inequality. It was strongly related to household food insecurity, children’s biological factors, and other immediate causes. These differences across countries support the idea that the same theoretical structure can appear differently based on welfare systems, health coverage, and levels of economic development.

The structural equation modelling (SEM) analysis in the study reveals a key difference in how SES may affect child malnutrition across two urban settings. In Hungary, a high-income setting, the theoretical model has less predictive power on malnutrition. Socioeconomic gradients have been greatly reduced due to strong social institutional protections, which is in line with prior studies in high-income countries and the developing countries of Brazil, Russia, India, China, and South Africa [49,50]. In Hungary, absolute deprivation is nearly nonexistent; for example, skipping meals, running low on food, or even experiencing hunger are rare and notable. Hungary is one of 25 countries with a Global Hunger Index (GHI) score of less than 5, which is low [51], and this makes the association between poverty and malnutrition largely ineffective. This aligns with a recent European health study that shows that access to universal healthcare, food security, and child welfare programs creates a protective effect that mitigates the association between household economic status and serious growth and health issues [52]. In contrast, in Indonesia, a rapidly urbanising LMIC, the same theoretical model shows strong predictive power. Similarly to previous studies in Indonesia, socioeconomic differences are the main predictors of nutritional inequality [39,53]. The findings from Indonesia support recent multi-country studies showing that in economies without robust social protections, household wealth directly affects access to food, healthcare, and the use of maternal knowledge, thereby increasing nutritional risks [54,55]. This contrast challenges the assumption that one-size-fits-all solutions apply to global nutrition and public health strategies, which underscores the need for models that account for local contexts.

### 4.2. Basic Causes: Socioeconomic Status and Wealth Status

The Hungarian model illustrates a high-income context in which both SES and wealth status protect children from the severe effects of urban material inequality on growth. The association between SES and malnutrition existed, but only within a narrow range of deprivation. Lower-SES households, then, still stand to gain from basic conditions that provide shelter against the most severe consequences of poor nutrition. In line with prior findings, wealth status, as material capital (e.g., housing and durable assets), was closely associated with other measures of socioeconomic security [56,57]. Nonetheless, the disparities in wealth did not lead to significant differences in child growth issues. This highlights findings from Europe over recent years and suggests that, as social policies and health systems have improved, the association between severe undernutrition and household income has weakened [58,59].

In Indonesia, basic socioeconomic conditions played a very different role. SES was a strong predictor of malnutrition. Wealth status indicators, such as asset ownership and housing conditions, increased the risk of poor nutrition. This trend aligns with recent studies from urban and peri-urban areas in LMICs, which find that economic hardship is the main cause of stunting and underweight condition. Previous studies from LMICs show that socioeconomic shocks [60], informal labour markets [61], and uneven social protection lead to rapid changes in nutritional vulnerability [62], depending on the economic position of the LMIC. The differences between the Hungarian and Indonesian models support the idea that the main causes of malnutrition are influenced by the overall institutional context. Welfare states lower, but do not completely remove, socioeconomic disparities, while transitional economies face direct and significant impacts.

### 4.3. Underlying Causes: Household Food Insecurity, Maternal Knowledge, and Household Environment

The second tier of the modified framework focuses on the underlying causes: household food insecurity, maternal nutrition knowledge, and household environment indicators. The relationship between SES and household food insecurity highlights that higher-SES families coexisted with food insecurity, yet this did not lead to increased malnutrition. Similar findings have appeared in other high-income European studies [63,64,65,66], where food insecurity shows as anxiety or dissatisfaction instead of actual undernourishment. Maternal nutrition knowledge appeared to be widely disseminated and was positively associated with child feeding practices in this study, supporting and protecting children from poor nutritional outcomes [67]. Furthermore, other studies indicate that maternal nutrition knowledge may improve dietary intake and dietary diversity, leading to better health and nutritional outcomes in preschoolers [68,69].

In Indonesia, household food insecurity is a major driver of child malnutrition, linking basic socioeconomic issues. Households with lower SES often struggle with food access, resulting in poor dietary quality and quantity. Food security cannot completely address the problems of food availability and affordability, while maternal nutrition knowledge is important. Evidence from other urban areas in LMICs aligns with this finding, indicating that barriers to maternal nutrition education vary by context, but several common issues persist. This is because even when mothers have better nutritional knowledge, household food insecurity makes it hard for them to follow recommended feeding practices [70]. Our findings show that nutrition education, particularly in Indonesia, should be combined with improvements in economic conditions and food systems to effectively reduce undernutrition [71].

Household food insecurity occurs when there is a lack of healthy and affordable food. It represents qualitative, quantitative, psychological, and social deprivations. This frequently occurs due to financial challenges, and prompts cravings among adults and children [72]. The current PLS-SEM analysis of urban preschoolers finds almost the opposite: this study observes near-sharp differences across countries in reliance on model- and data-based frameworks. In Hungary, there is both a high socio-economic level (95% of households are employed and all have health insurance) and very supportive family reinforcement across the surveyed households, yet they still experienced much food insecurity. Food insecurity links wealth to child factors and malnutrition. It is mostly a psychological strain related to food prices and the availability of better, healthier alternatives and food choices [73]. Interestingly, it does not appear to result in clinical undernutrition, as evidenced by the high proportion of individuals with normal nutritional status and very few cases of wasting or stunting. In Indonesia, where middle SES is widespread but socioeconomic realisation varies widely, food insecurity represents one of the strongest material drivers. It serves as a mediator through the limitation of maternal nutrition knowledge, poor household conditions, perinatal risks and inadequate feeding practices. Consequently, stunting, wasting, and underweight condition are much more prevalent.

The household environment, including factors such as parental education, income, and housing conditions, varies greatly across countries. In Hungary, that which is necessary to ensure basic nutrition for most children in the environment is closely tied to SES and wealth. These considerations showed a weak link to malnutrition. This suggests that the needs for improved living conditions and higher education levels have already been surpassed. As a result, the causes of malnutrition are becoming more complex and involve both clinical [74] and psychosocial factors [75]. In Indonesia, the household environment still plays a significant role in household food insecurity and in overall child health and nutrition outcomes. Recent studies indicate that neighbourhood infrastructure, sanitation, and household crowding are associated with these outcomes in preschoolers, including poor health and nutrition inequalities [53,76].

### 4.4. Immediate Causes: Child Biological Factors, Feeding Practices, Eating Behaviour, and Social Protection

The immediate causes of the framework include the child’s biological factors, feeding practices, eating behaviour, and social support through health coverage. The Hungarian model showed that children’s biological factors were associated with some intermediate variables, but they did not strongly predict malnutrition when broader structural conditions were considered. Early detection and treatment in well-funded healthcare systems across most European countries, including Hungary, ensure that prematurity and low birth weight are less likely to lead to chronic undernutrition. In Hungary, universal or nearly universal health coverage ensures that vulnerable infants receive follow-up care, growth monitoring, and nutritional counselling [77]. Recent evidence from high-income European countries shows that the long-term growth deficits linked to adverse birth outcomes can be significantly reduced when newborn and under-five children’s services are thorough. Children need access to preventive and primary healthcare without parents being discouraged by direct or out-of-pocket costs [78]. Furthermore, not all European Union countries protect children from the financial burden of unexpected ongoing healthcare costs for a more serious condition [79].

Child factors were moreover more strongly associated with malnutrition in Indonesia, which could be related to the cumulative effects of intrauterine growth retardation [80], premature birth [81], and early-life infections in less secure health environments [82]. Our results are consistent with the literature, which has repeatedly shown a strong relationship between low birth weight and prematurity as one of the strongest predictors of stunting and wasting in the absence of adequate neonatal and postnatal care [81]. These children’s biological vulnerabilities are less well buffered against communication from health services in Indonesia than in Hungary, and this potentially worsens due to household poverty and food insecurity.

In both studies, child feeding practices and eating behaviour were differentially important as predictors. Differences in complementary feeding, exclusive breastfeeding, and dietary habits did not distinguish malnourished from well-nourished children in Hungary. That may be due to the frequent availability of calorie-rich foods, common nutrition advisories, and ongoing paediatric screening for growth. This decreases the likelihood that failed feeding practices will lead to observable undernutrition. By contrast, the feeding practices in Indonesia were more constrained by material conditions, thereby becoming intertwined with SES and household food insecurity; the direct effect of feeding practices on malnutrition was attenuated. This is consistent with recent studies indicating that interventions focused solely on counselling or behaviour change face limited success in severely resource-limited settings unless living standards improve concurrently [83].

Health coverage, as a modality of social and health protection, was shown to be a protective health programme in both countries, but effects and approaches differed. Hungary has close to universal health insurance, and most children are monitored for growth problems, resulting in early identification of those with difficulties so that the effects of socioeconomic differences can be contained relative to risks for malnutrition. In Hungary, the broad accessibility of services may obscure differences in health coverage. This study found that health differences across socioeconomic groups might be better explained by household resources and food access than by insurance status. This occurs in part because health access is deeply intertwined with economic conditions [50]. Nevertheless, social protection can help those affected to avoid risks, provide timely care, and reduce the costs of illness [78]. Health coverage in Indonesia was found to be related to SES and to mediate the effect of poverty on child health. Individuals with higher SES preferred more extensive insurance coverage, as well as more dependable services and high-quality facilities. This created a self-reinforcing cycle of income-based social protection. Other LMICs implementing universal schemes also show inequities in coverage, utilisation and quality of care [84]. The challenges for universal health coverage in LMICs are that health insurance is not available for low-SES households, with millions of low-income children and parents working in the informal sector forced to choose between health care and other basic needs [85].

### 4.5. Comparative Structural Pathways and Multi-Group Analysis

The PLS-SEM modelling method permitted a nuanced treatment of the basic causes, specifically separating the levels of SES and wealth. The modelling in both countries distinguished between SES (the theory of human capital, such as education and employment) and wealth [86], which is the accumulation of material capital. These analyses demonstrated that these factors represent partially overlapping, but also non-redundant dimensions of social position [87]. Based on a prior study in Hungary, SES was more strongly associated with health knowledge and access [88]. Furthermore, the current findings indicate that these less strongly correlated indicators of wealth were associated with lifestyle amenities that were also more weakly correlated with malnutrition. This finding is consistent with a previous study that found that wealth was most strongly associated with shelter and food security in Indonesia, where it was linked to food insecurity as part of the measurement of material deprivation, which may finally impact child nutrition outcomes [89].

This study confirms earlier theoretical explanations that postulate that temporary income, assets, and stored goods have different impacts on undernutrition and supports recent evidence that wealth-related choices can be stronger predictors of long-term well-being outcomes because they reflect, at least in part, a household’s longer-run economic viability [90]. Therefore, in a cross-national study of child malnutrition, it is important to treat SES and wealth as different but interrelated drivers [91]. The MGA demonstrated the complexity of this pathway; in the two countries, SES operates through two completely different pathways [92]. Although the mechanisms explaining how child feeding practices relate to growth outcomes are largely similar across settings and specific behaviours, the determinants of these practices differ widely by geography. SES has a direct effect on undernutrition in Hungary, mainly through its indirect influence on maternal nutrition knowledge and the accessibility of health services. In contrast, SES serves as a fundamental structural driver in Indonesia, with attributable impacts on the burden of malnutrition, household food security, and maternal knowledge [93]. These comparisons demonstrate the utility of multi-group SEM for separating intractable biological facts from context-specific SES.

These divergent roles of SES in structural models mirror the larger epidemiological and nutritional contexts of both countries. Nutritional problems, such as undernutrition, which are low-prevalence and weakly stratified factors in Hungary, also show similar trends across Europe, with growth deficits becoming increasingly rare, and almost invariably limited to clinical subpopulations suffering from chronic illness or complex psychosocial adversity [94]. This study is conducted with caution, as our predominantly urban sample likely underrepresents structurally marginalised groups, including those residing in deep poverty, where nutritional inequities are particularly persistent [95]. By contrast, the socioeconomic gradients evident in the Indonesian sample are well aligned with the persistent features of Southeast Asian cities that continue to capture many of the displacements of rapid urbanisation [96]. While recent economic development in Indonesia has begun to change its dietary patterns, even leading to an increase in diet-related non-communicable diseases among higher-income segments, our results show that broad macroeconomic progress is insufficient to eliminate the underlying structural disadvantages faced by low-income households residing in urban areas [16].

Overall, while PLS-SEM path analysis in this study revealed significant relationships regarding household food insecurity and SES, we caution against over-interpreting these results, as they should be considered in light of the model’s explanatory power. While R^2^ values for child malnutrition were low in Indonesia (0.024) and Hungary (0.062), predictive relevance (Q^2^) stayed close to zero or negative at any single value of the model. To conclude, these numbers show that although the socioeconomic and behavioural pathways modelled in this study are statistically significant, they explain only a small proportion of the total variance in malnutrition. The few predictive values are limited due to the extreme complexity and multifactorial nature of child undernutrition. This implies that malnutrition in these study populations is probably mostly determined by unmeasured factors, such as immediate biological determinants, infectious disease burden, genetic predispositions and environmental sanitation (WASH). Hence, this model should be seen as the highlighting of certain socioeconomic pathways for risk rather than a fully predictive model.

### 4.6. Implications for Policy and Future Research

The cross-national contrast that this study provides emphasises the importance of context-specific dietary policy and warns against the uncritical transfer of nutritional programs across diverse settings. Broad universalist poverty alleviation is likely to be diminishing in its returns at the low levels of child undernutrition currently present in Hungary, and policy should shift towards prioritising clinical and social targets, rather than increasing the need for support. This implies that, in cases of malnutrition in high-income settings, the simultaneous consideration of psychological, clinical, and social work characteristics would be well advised. In contrast, the Indonesian data further emphasise that child undernutrition remains mainly attributable to SES, and that solutions require macroeconomic actions. In Indonesia, scaling up social protection and bundled cash transfers with nutrition-specific counselling are essential for improving child growth. In methodological terms, the study operationalises the modified UNICEF approach using SEM, which shows that while malnutrition is ultimately driven by similar variables across socioeconomic contexts, these drivers predict malnutrition risk to widely varying extents. Future studies should use this multi-group modelling strategy in other countries and in rural populations to disentangle how different welfare states, availability of healthcare services and patterns of urbanisation affect the association between SES and child nutrition.

### 4.7. Strengths and Limitations

This study has further methodological strengths that provide confidence in the findings and cross-national comparability. Complex relationships with formative constructs, including sociodemographic factors such as income, education, occupational status, and perceived personal wealth, were studied between the preparatory variables and the outcome. PLS-SEM with MGA was deemed appropriate. PLS-SEM was also appropriate for handling the (smaller) Hungarian and (larger) Indonesian datasets, as it can handle non-normal data and multiple latent variables. The reliability and validity of all constructs were established through validation, using VIF, HTMT, outer loadings, AVE, and composite reliability. Moreover, the MICOM of partial measurement invariance, allowing for proper comparison between Hungary and Indonesia, was demonstrated. Compared to other designs, comparative designs better revealed divergent socioeconomic pathways in high-income and lower-middle-income settings, and distal socioeconomic factors, intermediate mechanisms, and proximal determinants jointly characterised the multilevel structure that shaped child malnutrition.

However, some limitations need to be recognised despite these strengths. The study’s cross-sectional design does not support causal inference, as it is impossible to establish temporal precedence among SES, food insecurity, maternal knowledge and feeding practices, and malnutrition. Although sufficient for PLS-SEM, recruitment from a single urban centre may limit the power of the analysis to detect smaller effects and their generalisability, particularly as to rural areas or other regions. The potential selection bias may limit comparisons of absolute prevalence rates across countries due to differences in sampling frames (kindergartens in Hungary versus Posyandu in Indonesia), but this is mitigated by its focus on internal relational pathways. The Hungarian sample had virtually no low-SES representation, which limits predictor variability and potentially leads to unstable path estimates, making it more difficult to directly compare socioeconomic impacts on malnutrition across countries. Self-reported data may also be affected by recall and social desirability biases, especially with food insecurity, maternal knowledge, subjective wealth and feeding practices. Moreover, anthropometry for malnutrition identifies stunting, wasting, and underweight, but not micronutrient deficiencies, dietary quality, or cognitive outcomes.

While PLS-SEM is a robust analysis, the relatively small sample size in Hungary (*n* = 128 versus *n* = 535 in Indonesia) and the unequal sample sizes could destabilise parameter estimates in the smaller group and lower the statistical power to detect differences in structural pathways. This means that comparative results must be treated cautiously, given the increased risk of Type II errors, and this might call for a more balanced, well-powered sample in future cross-cultural studies. Future studies would also benefit from longitudinal and multi-site designs, objective nutritional and biological indicators of mother-to-child transmission, and validated interventions for food insecurity. Finally, intervention studies testing whether addressing identified mediators, such as food insecurity and maternal nutrition knowledge, would reduce socioeconomic gradients in malnutrition would translate structural insights into actionable policy.

## 5. Conclusions

The comparative multigroup SEM analysis does not support the idea that socioeconomic inequalities in childhood malnutrition are worse in urban Indonesia than in Hungary. In Indonesia, this relationship is linked by various factors, including early-life health, household food insecurity, maternal nutritional knowledge, and access to healthcare. This suggests that nutritional outcomes are primarily associated with systemic resource constraints rather than individual behaviours. In Hungary, a wealthy country with established social safety nets and healthcare systems, socioeconomic inequalities are less severe but still exist through non-material paths, such as maternal knowledge, stress, and health-related behaviours. These findings reveal important differences in how structural socioeconomic factors relate to nutritional outcomes. However, we must recognise the model’s limited predictive power, as shown by the low R^2^ and Q^2^ values. Nevertheless, caution should be exercised when interpreting these findings, by virtue of several important study limitations. First, we are not sure that the context between these two countries (Hungary and Indonesia) has full comparability of construct, which can affect the multi-group comparison. In addition, it is important to note that the model has limited predictive performance for malnutrition, since R^2^ and Q^2^ values were low. This highlights that childhood malnutrition is a complex issue, one associated mainly with unmeasured biological, environmental, and health factors beyond the socioeconomic and behavioural aspects explored in this study.

## Figures and Tables

**Table 1:** Variance Inflation Factor (VIF) for Hungarian and Indonesian model (Source: Primary Data Survey).

Variables	Hungary		Indonesia	
	VIF	Decision	VIF	Decision
Child eating behaviour	1	Valid	1	Valid
Complementary feeding	1.913	Valid	1.056	Valid
Education level	1.068	Valid	1.137	Valid
Electric car ownership	2.213	Valid	1.686	Valid
Ever breastfeeding	1.412	Valid	-	-
Exclusive breastfeeding	2.589	Valid	1.187	Valid
Expensive food	2.642	Valid	1.981	Valid
Household food insecurity	1	Valid	1	Valid
Family income	1.068	Valid	1.137	Valid
Initiation of breastfeeding	1.278	Valid	-	-
Low birth weight	1.174	Valid	1	Valid
Maternal nutrition knowledge	1	Valid	1	Valid
Mother age	1.07	Valid	-	-
Nanny/servant	2.215	Valid	1.829	Valid
Prematurity	1.174	Valid	-	
Private school	2.275	Valid	2.057	Valid
SES	1	Valid	1	Valid
Travel abroad	2.213	Valid	-	-
Weaning	1.81	Valid	1.164	Valid
Health assurance	-	-	1	Valid
Feeding frequency	-	-	1.01	Valid
Car ownership	-	-	1.159	Valid
Child’s gender	-	-	1	Valid
Malnutrition	1	Valid	1	Valid

VIF < 3 = valid.

**Table 2:** HTMT between SES and wealth status in the study (*n* = 128, Hungary; *n* = 535, Indonesia; Source: Primary Data Survey, PLS-SEM test).

Construct 1	Construct 2	HTMT Value	Decision
Hungary: SES	Wealth status	0.445	Pass
Indonesia: SES	Wealth status	0.025	Pass

HTMT < 0.85 = Pass.

**Table 3:** Model fit indices for the modified structural model (*n* = 128, Hungary; *n* = 535, Indonesia; Source: Primary Data Survey, PLS-SEM test).

Rule of Thumb	Hungary	Decision	Indonesia	Decision
SRMR	0.090	Fit	0.078	Fit
Q^2^ Malnutrition	−0.009	Less predictive	0.016	Predictive
Q^2^ SES	0.564	Strong predictive	0.409	Strong predictive
Q^2^ Child Factors	0.002	Weak predictive	0.017	Weak predictive
Q^2^ Maternal Nutrition Knowledge	0.091	Moderate predictive	0.038	Moderate predictive
Q^2^ Household Food Insecurity	0.013	Weak predictive	0.044	Moderate predictive
Q^2^ Child Feeding Practices	0.027	Moderate predictive	0.024	Moderate predictive
Q^2^ Child Eating Behaviour	−0.001	Less predictive	−0.003	Less predictive
Q^2^ Wealth Status	0.129	Moderate predictive	0.283	Strong predictive

SRMR < 0.10 = fit; Q^2^ > 0 = predictive; Q^2^ < 0 = less predictive; Q^2^ < 0.02 = weak; Q^2^ < 0.15 = Moderate; Q^2^ < 0.35 = Strong.

**Table 4:** Mediation effects (standardised) on child malnutrition in the structural modelling for Hungary (*n* = 128, Hungary; Source: Primary Data Survey, PLS-SEM test).

Latent Variables	*p* Value			Mediation Analysis
	Direct Effect	Indirect Effect	Total Effect	
Socioeconomic Status	S *	NS	S *	No mediation
Child Factors	NS	NS	NS	Not applicable
Child Eating Behaviour	NS	NS	NS	Not applicable
Child Feeding Practices	NS	NS	NS	Not applicable
Household Food Insecurity	S **	NS	S **	No mediation
Health Coverage	-	-	-	Not applicable
Household Environment	NS	S *	S *	Full mediation
Maternal Nutrition Knowledge	NS	NS	NS	Not applicable
Wealth Status	-	S *	S *	Full mediation

* *p* < 0.05, ** *p* < 0.01; NS, not significant; S, significant.

**Table 5:** Mediation path analysis on child malnutrition in Hungary, as included in the study (*n* = 128, Hungary; Source: Primary Data Survey, PLS-SEM test).

Indirect Effect Paths	S (β)	SD	T Statistics	*p* Values	Decision
Household Environment → SES → Malnutrition	0.156	0.072	2.175	0.030	S
Household Environment → SES → Maternal Nutrition Knowledge	0.242	0.043	5.619	0.000	S
Household Environment → Wealth Status → Child Eating Behaviour	−0.053	0.025	2.095	0.036	S
Household Environment → Wealth Status → Food Insecurity	0.078	0.019	4.123	0.000	S
Wealth Status → Food Insecurity → Child Factors	−0.016	0.007	2.377	0.017	S
Wealth Status → Food Insecurity → Malnutrition	−0.020	0.008	2.594	0.010	S
Household Environment → Wealth Status → Food Insecurity → Malnutrition	−0.007	0.003	2.470	0.014	S

S (β), standardized coefficient; SD, standard deviation; *p* value < 0.05 = significant; →, refers.

**Table 6:** Mediation effects (standardised) on child malnutrition in the structural modelling for Indonesia (*n* = 535, Indonesia; Source: Primary Data Survey, PLS-SEM test).

Latent Variables	*p* Value			Mediation Analysis
	Direct Effect	Indirect Effect	Total Effect	
Socioeconomic Status	S **	S *	S **	Partial mediation
Child Factors	S *	NS	S **	No mediation
Child Eating Behaviour	NS	NS	NS	Not applicable
Child Feeding Practices	NS	NS	NS	Not applicable
Household Food Insecurity	NS	NS	NS	Not applicable
Health Coverage	NS	NS	NS	Not applicable
Household Environment	NS	S **	S **	Full mediation
Maternal Nutrition Knowledge	NS	NS	NS	Not applicable
Wealth Status	NS	NS	NS	Not applicable

* *p* < 0.05, ** *p* < 0.01; NS, not significant; S, significant.

**Table 7:** Mediation path analysis on child malnutrition in Indonesia, as included in the study (*n* = 535, Indonesia; Source: Primary Data Survey, PLS-SEM test).

Indirect Effect Paths	S (β)	SD	T Statistics	*p* Values	Decision
Household Environment → SES → Child Factors	0.084	0.027	3.062	0.002	S
Household Environment → SES → Health Coverage	0.089	0.024	3.659	0.000	S
Household Environment → SES → Malnutrition	0.073	0.026	2.770	0.006	S
Household Environment → SES → Maternal Nutrition Knowledge	0.092	0.025	3.743	0.000	S
Household Environment → Wealth Status → Food Insecurity	0.114	0.023	5.001	0.000	S
Household Environment → Wealth Status → SES	0.047	0.023	2.032	0.042	S
Household Environment → SES → Child Factors → Malnutrition	0.009	0.005	1.864	0.049	S

S (β), standardized coefficient; SD, standard deviation; *p* value < 0.05 = significant; →, refers; S, significant.

**Table 8:** The results of MICOM Step 2 between Hungary and Indonesia (*n* = 128, Hungary; *n* = 535, Indonesia; Source: Primary Data Survey, PLS-SEM test).

Variables	Original Correlation	Correlation Permutation Mean	5.00%	Permutation *p*-Value	Original Correlation	Decision
Child Eating Behaviour	1	1	1	0.387	1	Established
Food Insecurity	1	1	1	0.414	1	Established
Malnutrition	1	1	1	0.376	1	Established
Maternal Nutrition Knowledge	1	1	1	0.327	1	Established
SES	1	1	1	0.028	1	Not established

*p*-value < 0.05 = not established.

**Table 9:** Permutation values in Hungary and Indonesia (*n* = 128, Hungary; *n* = 535, Indonesia; Source: Primary Data Survey, PLS-SEM test).

Variables	Original Difference	Permutation Mean Difference	2.50%	97.50%	Permutation *p*-Value	Decision
Child Eating Behaviour	−1.795	−0.009	−0.164	0.133	0.000	Not established
Food Insecurity	−1.664	−0.008	−0.169	0.127	0.000	Not established
Malnutrition	−0.882	−0.007	−0.08	0.035	0.000	Not established
Maternal Nutrition Knowledge	0.239	−0.013	−0.303	0.253	0.092	Established
SES	0.144	−0.015	−0.345	0.285	0.370	Established

*p*-value < 0.05 = not established.

**Table 10:** Results of multi-group analysis between Hungary and Indonesia (*n* = 128, Hungary; *n* = 535, Indonesia; Source: Primary Data Survey, PLS-SEM test).

Path	Difference (Hungary-Indonesia)	*p* Value (Hungary-Indonesia)	Decision
Child Eating Behaviour → Malnutrition	−0.029	0.768	Not significant difference
Food Insecurity → Child Eating Behaviour	−0.052	0.324	Not significant difference
Food Insecurity → Malnutrition	−0.178	0.120	Not significant difference
Maternal Nutrition Knowledge → Child Eating Behaviour	−0.044	0.394	Not significant difference
Maternal Nutrition Knowledge → Food Insecurity	−0.068	0.582	Not significant difference
Maternal Nutrition Knowledge → Malnutrition	−0.040	0.778	Not significant difference
SES → Child Eating Behaviour	0.131	0.677	Not significant difference
SES → Food Insecurity	0.012	0.001	Significant difference
SES → Malnutrition	0.100	0.001	Significant difference
SES → Maternal Nutrition Knowledge	0.141	0.000	Significant difference

*p* value < 0.05 = significant.

## Data Availability

The raw data of this study are available upon request to the authors for the purpose of academic research.

## Abbreviations

The following abbreviations are used in this manuscript:

AVE	Average Variance Extracted
CEBQ	Child Eating Behaviour Questionnaire
CR	Cronbach’s Alpha
FIES-SM	Food Insecurity Experience Scale Survey Module
GHI	Global Hunger Index
GNKQ-R	General Nutrition Knowledge Questionnaire–Revised
HAZ	Height-for-Age
HTMT	Heterotrait–Monotrait
IYCF	Infant and Young Child Feeding
LMICs	Low- and Middle-Income Countries
LPA	Latent Profile Analysis
MGA	Multi-Group Analysis
MICOM	Measurement Invariance of Composite Models
PLS-SEM	Partial Least Squares Structural Equation Modelling
Q2	Predictive Relevance
S (β)	Standardized Coefficient
SD	Standard Deviation
SDGs	Sustainable Development Goals
SEM	Structural Equation Modelling
SES	Socioeconomic status
SRMR	Standardised Root Mean Square Residual
UNICEF	United Nations International Children’s Emergency Fund
VIF	Variance Inflation Factors
WAZ	Weight-for-Age
WHO	World Health Organisation
WHZ	Weight-for-Height

## Appendix A

### Appendix A.1

**Table A1:** Data respondents’ characteristics in Hungary and Indonesia, as included in the study (*n* = 128, Hungary; *n* = 535, Indonesia; Source: Primary Data Survey).

Predictors	Hungary Malnutrition *n* (%); 15 (11.7)	Hungary Non-Malnutrition *n* (%); 113 (88.3)	Indonesia Malnutrition *n* (%); 268 (50.1)	Indonesia Non-Malnutrition *n* (%); 267 (49.9)
Child’s gender				
Boy	9 (12.7%)	62 (87.3%)	149 (55.0%)	122 (45.0%)
Girl	6 (10.5%)	51 (89.5%)	119 (45.1%)	145 (54.9%)
Prematurity				
Yes	2 (33.3%)	4 (66.7%)	131 (53.3%)	115 (46.7%)
No	13 (10.7%)	109 (89.3%)	137 (47.4%)	152 (52.6%)
Low birth weight				
Yes	2 (50.0%)	2 (50.0%)	31 (62.0%)	19 (38.0%)
No	13 (10.5%)	111 (89.5%)	237 (48.9%)	248 (51.1%)
Socioeconomic status				
Low	0 (0.0%)	0 (0.0%)	19 (70.4%)	8 (29.6%)
Middle	5 (27.8%)	13 (72.2%)	241 (50.3%)	238 (49.7%)
High	10 (9.1%)	100 (90.9%)	8 (27.6%)	21 (72.4%)
Income per month				
≤HUF 230,000 or ≤IDR 2,300,000	1 (100.0%)	0 (0.0%)	116 (55.2%)	94 (44.8%)
HUF 230,001–450,000 or IDR 2,300,001–4,500,000	1 (10.0%)	9 (90.0%)	115 (48.7%)	121 (51.3%)
HUF 450,001–570,000 or IDR 4,500,001–5,700,000	2 (28.6%)	5 (71.4%)	22 (41.5%)	31 (58.5%)
HUF 570,001–700,000 or IDR 5,700,001–7,000,000	2 (11.8%)	15 (88.2%)	10 (47.6%)	11 (52.4%)
HUF 700,001–1,000,000 or IDR 7,000,001–10,000,000	2 (5.1%)	37 (94.9%)	4 (44.4%)	5 (55.6%)
>HUF 1,000,001 or >IDR 10,000,001	7 (13.0%)	47 (87.0%)	1 (16.7%)	5 (83.3%)
Maternal education				
Low	0 (0.0%)	2 (100.0%)	90 (60.0%)	60 (40.0%)
Moderate	11 (14.1%)	67 (85.9%)	143 (47.0%)	161 (53.0%)
High	4 (8.3%)	44 (91.7%)	35 (43.2%)	46 (56.8%)
Mother’s age:				
<20 years	1 (14.3%)	6 (85.7%)	1 (100.0%)	0 (0.0%)
≥20–29 years	2 (5.7%)	33 (94.3%)	94 (55.0%)	77 (45.0%)
≥30–39 years	12 (14.0%)	74 (86.0%)	126 (45.5%)	151 (54.5%)
≥40–49 years	0 (0.0)	0 (0.0)	47 (54.7%)	39 (45.3%)
Employment status				
Employed	15 (12.6%)	104 (87.4%)	110 (49.1%)	114 (50.9%)
Unemployed	0 (0.0%)	9 (100.0%)	158 (50.8%)	153 (49.2%)
Subjective wealth status				
rather better off	0 (0.0%)	1 (100.0%)	73 (45.6%)	87 (54.4%)
average	9 (10.2%)	79 (89.8%)	184 (51.0%)	177 (49.0%)
rather worse off	6 (15.4%)	33 (84.6%)	11 (78.6%)	3 (21.4%)
House ownership				
Yes	11 (10.1%)	98 (89.9%)	88 (49.4%)	90 (50.6%)
No	4 (21.1%)	15 (78.9%)	180 (50.4%)	177 (49.6%)
Car ownership				
Yes	15 (12.2%)	108 (87.8%)	30 (42.9%)	40 (57.1%)
No	0 (0.0%)	5 (100.0%)	238 (51.2%)	227 (48.8%)
Health insurance				
Yes	15 (11.7%)	113 (88.3%)	145 (50.7%)	141 (49.3%)
No	0 (0.0)	0 (0.0)	123 (49.4%)	126 (50.6%)
Family size				
Small (≤3 members)	4 (14.8%)	23 (85.2%)	46 (50.5%)	45 (49.5%)
Medium (4–6 members)	10 (10.2%)	88 (89.8%)	186 (50.3%)	184 (49.7%)
Large (>6 members)	1 (33.3%)	2 (66.7%)	36 (48.6%)	38 (51.4%)
Region (Indonesia)				
Central	-	-	37 (52.1%)	34 (47.9%)
East	-	-	78 (52.3%)	71 (47.7%)
North	-	-	65 (43.3%)	85 (56.7%)
South	-	-	68 (54.4%)	57 (45.6%)
West	-	-	20 (50.0%)	20 (50.0%)
Region (Hungary)				
Pest	13 (14.3%)	78 (85.7%)	-	-
Buda	2 (5.4%)	35 (94.6%)	-	-
Under-five sibling:				
≤2 children	14 (11.1%)	112 (88.9%)	253 (50.0%)	253 (50.0%)
≥3 children	1 (50.0%)	1 (50.0%)	15 (51.7%)	14 (48.3%)
1. Child feeding practices				
Age at starting complementary feeding				
Less than 6 months	4 (10.8%)	33 (89.2%)	25 (53.2%)	22 (46.8%)
6 months	10 (11.9%)	74 (88.1%)	202 (49.1%)	209 (50.9%)
More than 6 months	1 (14.3%)	6 (85.7%)	41 (53.2%)	36 (46.8%)
Feeding frequency				
2 times	1 (33.3%)	2 (66.7%)	47 (54.0%)	40 (46.0%)
2–3 times	3 (10.7%)	25 (89.3%)	156 (48.9%)	163 (51.1%)
4–6 times and more	11 (11.3%)	86 (88.7%)	65 (50.4%)	64 (49.6%)
Initiation breastfeeding:				
Within 1 h	10 (11.2%)	79 (88.8%)	76 (43.2%)	100 (56.8%)
After 1 h or more	5 (12.8%)	34 (87.2%)	192 (53.5%)	167 (46.5%)
Ever breastfeeding				
Yes	14 (11.9%)	104 (88.1%)	245 (49.4%)	251 (50.6%)
No	1 (10.0%)	9 (90.0%)	23 (59.0%)	16 (41.0%)
Exclusive breastfeeding				
Yes	9 (11.7%)	68 (88.3%)	97 (47.5%)	107 (52.5%)
No	6 (11.8%)	45 (88.2%)	171 (51.7%)	160 (48.3%)
Baby weaning				
Less than 6 months	5 (14.7%)	29 (85.3%)	53 (52.5%)	48 (47.5%)
Between 6 months and 24 months	8 (11.9%)	59 (88.1%)	75 (47.2%)	84 (52.8%)
24 months or more	2 (7.4%)	25 (92.6%)	140 (50.9%)	135 (49.1%)
2. Household food insecurity				
Food-secure	15 (12.3%)	107 (87.7%)	155 (46.8%)	176 (53.2%)
Food-insecure	0 (0.0%)	6 (100.0%)	113 (55.4%)	91 (44.6%)
3. Maternal nutrition knowledge				
Low	0 (0.0%)	1 (100.0%)	75 (51.7%)	70 (48.3%)
Moderate	8 (12.7%)	55 (87.3%)	190 (49.2%)	196 (50.8%)
High	7 (10.9%)	57 (89.1%)	3 (75.0%)	1 (25.0%)
4. Child eating behaviour				
Food-approach				
- Typical/balance eater	7 (11.7%)	53 (88.3%)	66 (53.2%)	58 (46.8%)
- Avid eater	0 (0.0%)	2 (100.0%)	71 (48.3%)	76 (51.7%)
Food-avoidant				
- Picky/fussy eater	8 (12.1%)	58 (87.9%)	18 (40.0%)	27 (60.0%)
- Poor eater	0 (0.0)	0 (0.0)	113 (51.6%)	106 (48.4%)

### Appendix A.2

**Table A2:** Outer loading validation of the latent variables in Hungary and Indonesia included in the study (*n* = 128, Hungary; *n* = 535, Indonesia; Source: Primary Data Survey, PLS-SEM Test).

Latent Variable	Indicators	Outer Loading	
		Hungary	Indonesia
Child Factor	Child’s gender	−0.038/invalid	0.768/valid
	Low birth weight	0.779/valid	0.602/valid
	Prematurity	0.873/valid	0.305/invalid
Wealth Status	Car ownership	0.293/invalid	0.571/valid
	House/villa ownership	0.386/invalid	0.381/invalid
	Subjective wealth status	−0.625/invalid	0.238/invalid
	Affordability of an electric car	0.762/valid	0.637/valid
	Affordability of expensive food	0.684/valid	0.691/valid
	Affordability nanny/servant	0.776/valid	0.694/valid
	Affordability of private school	0.714/valid	0.734/valid
	Affordability of travel abroad	0.654/valid	0.491/valid
	Affordability of wired internet	0.056/invalid	0.451/invalid
	Existence of living room	0.301/invalid	0.421/invalid
	Existence of separate bathroom	-	0.381/invalid
	Existence of separate kitchen	0.158/invalid	0.379/invalid
	Existence of sport/hobby room	0.239/invalid	0.335/invalid
Household Environment	Education level	0.564/valid	0.772/valid
	Employment	0.027/invalid	0.188/invalid
	Family size	−0.367/invalid	0.128/invalid
	Family Income	0.829/valid	0.857/valid
	Mother’s age	0.538/valid	−0.080/invalid
	Under-five sibling	−0.125/invalid	0.162/invalid
	Region	0.198/invalid	−0.037/invalid
Child Feeding Practices	Complementary feeding	0.695/valid	0.540/valid
	Ever breastfeeding	0.658/valid	0.176/valid
	Exclusive breastfeeding	0.830/valid	0.676/valid
	Feeding frequency	0.210/invalid	0.517/valid
	Initiation breastfeeding	0.565/valid	−0.154/invalid
	Weaning practices	0.817/valid	0.534/valid

Outer loading > 0.50, valid.

### Appendix A.3

**Table A3:** Reliability and validity tests of constructs and model fit statistics for the measurement model (*n* = 128, Hungary; Source: Primary Data Survey, PLS-SEM Test).

Constructs/Latent Variables	CR	√AVE	Decision
Child factors	0.816	0.831	Valid and reliable
Wealth status	0.913	0.822	Valid and reliable
Household environment	0.702	0.671	Valid and reliable
Child feeding practices	0.842	0.723	Valid and reliable

AVE, average variance extracted; CR, composite reliability.

### Appendix A.4

**Table A4:** Reliability and validity tests of constructs and model fit statistics for the measurement model for Indonesia (*n* = 535, Indonesia; Source: Primary Data Survey, PLS-SEM Test).

Constructs/Latent Variables	CR	√AVE	Decision
Child factors	0.665	0.710	Valid and reliable
Wealth status	0.851	0.598	Valid and reliable
Household environment	0.802	0.819	Valid and reliable
Child feeding practices	0.690	0.701	Valid and reliable

AVE, average variance extracted; CR, composite reliability.

### Appendix A.5

**Table A5:** Inner model fit using *R^2^* for Hungary (*n* = 128, Hungary; *n* = 535, Indonesia; Source: Primary Data Survey).

Variable		Hungary			Indonesia	
	R^2^	R^2^ Adjusted	Decision	R^2^	R^2^ Adjusted	Decision
Child Eating Behaviour	0.073	0.050	Weak	0.016	0.005	Weak
Child Factors	0.040	0.032	Weak	0.020	0.016	Weak
Child Feeding Practices	0.126	0.109	Weak	0.043	0.034	Weak
Food Insecurity	0.057	0.050	Weak	0.054	0.050	Weak
Health Coverage	-	-	-	0.022	0.021	Weak
Malnutrition	0.084	0.062	Weak	0.037	0.024	Weak
Maternal Nutrition Knowledge	0.091	0.084	Weak	0.026	0.022	Weak
SES	0.579	0.576	Moderate	0.418	0.416	Moderate
Wealth Status	0.141	0.138	Weak	0.292	0.290	Moderate

R^2^ < 0.25 = weak; 0.25 < R^2^ < 0.50 = moderate; R^2^ > 0.75 = strong [97].

### Appendix A.6

**Table A6:** Standardised and unstandardised estimates for direct effects on the structural model for Hungary (*n* = 128, Hungary; Source: Primary Data Survey, PLS-SEM test).

Direct Effect Paths	S (β)	SD	T Statistics	*p* Values	Decision
Child Eating Behaviour → Malnutrition	0.008	0.057	0.143	0.887	NS
Child Factors → Child Eating Behaviour	−0.137	0.045	3.038	0.002	S
Child Factors → Malnutrition	0.176	0.116	1.519	0.129	NS
Child Feeding Practices → Child Eating Behaviour	0.075	0.071	1.057	0.291	NS
Child Feeding Practices → Malnutrition	−0.019	0.059	0.321	0.748	NS
Food Insecurity → Child Eating Behaviour	−0.134	0.088	1.524	0.128	NS
Food Insecurity → Child Factors	−0.078	0.026	2.942	0.003	S
Food Insecurity → Child Feeding Practices	−0.064	0.058	1.118	0.264	NS
Food Insecurity → Malnutrition	−0.095	0.028	3.379	0.001	S
Food Insecurity → Maternal Nutrition Knowledge	−0.041	0.048	0.846	0.398	NS
Household Environment → Child Feeding Practices	0.121	0.125	0.974	0.330	NS
Household Environment → SES	0.796	0.038	21.044	0.000	S
Household Environment → Wealth Status	0.376	0.048	7.887	0.000	S
Maternal Nutrition Knowledge → Child Eating Behaviour	−0.099	0.068	1.460	0.144	NS
Maternal Nutrition Knowledge → Child Feeding Practices	0.290	0.077	3.768	0.000	S
Maternal Nutrition Knowledge → Malnutrition	−0.042	0.062	0.674	0.500	NS
SES → Child Eating Behaviour	0.171	0.060	2.862	0.004	S
SES → Child Factors	0.194	0.107	1.803	0.071	NS
SES → Child Feeding Practices	−0.027	0.101	0.263	0.793	NS
SES → Food Insecurity	0.084	0.085	0.982	0.326	NS
SES → Malnutrition	0.196	0.088	2.215	0.027	S
SES → Maternal Nutrition Knowledge	0.304	0.050	6.024	0.000	S
Wealth Status → Child Eating Behaviour	−0.142	0.062	2.273	0.023	S
Wealth Status → Child Feeding Practices	0.063	0.073	0.868	0.385	NS
Wealth Status → Food Insecurity	0.209	0.045	4.670	0.000	S
Wealth Status → SES	−0.111	0.051	2.166	0.030	S

S (β), standardized coefficient; SD, standard deviation; *p* value < 0.05, significant; →, refers; S, significant; NS, Not Significant.

### Appendix A.7

**Table A7:** Standardised and unstandardised estimates for direct effects on the structural model for Indonesia included in the study (*n* = 535, Indonesia; Source: Primary Data Survey, PLS-SEM test).

Direct Effect Paths	S (β)	SD	T Statistics	*p* Values	Decision
Child Eating Behaviour → Malnutrition	0.006	0.042	0.136	0.892	NS
Child Factors → Child Eating Behaviour	0.079	0.049	1.623	0.105	NS
Child Factors → Malnutrition	0.107	0.047	2.301	0.021	S
Child Feeding Practices → Child Eating Behaviour	−0.067	0.059	1.138	0.255	NS
Child Feeding Practices → Malnutrition	0.023	0.049	0.475	0.635	NS
Food Insecurity → Child Eating Behaviour	−0.047	0.045	1.041	0.298	NS
Food Insecurity → Child Factors	−0.008	0.057	0.135	0.892	NS
Food Insecurity → Child Feeding Practices	0.021	0.055	0.387	0.699	NS
Food Insecurity → Malnutrition	0.070	0.043	1.614	0.107	NS
Food Insecurity → Maternal Nutrition Knowledge	0.024	0.044	0.550	0.582	NS
Health Coverage → Malnutrition	−0.039	0.044	0.875	0.382	NS
Household Environment → Child Feeding Practices	0.227	0.075	3.030	0.002	S
Household Environment → SES	0.596	0.038	15.636	0.000	S
Household Environment → Wealth Status	0.540	0.039	13.928	0.000	S
Maternal Nutrition Knowledge → Child Eating Behaviour	−0.039	0.043	0.900	0.368	NS
Maternal Nutrition Knowledge → Child Feeding Practices	0.062	0.058	1.082	0.279	NS
Maternal Nutrition Knowledge → Malnutrition	−0.023	0.043	0.522	0.602	NS
SES → Child Eating Behaviour	−0.037	0.049	0.766	0.444	NS
SES → Child Factors	0.141	0.043	3.265	0.001	S
SES → Child Feeding Practices	−0.018	0.082	0.222	0.824	NS
SES → Food Insecurity	0.042	0.040	1.031	0.303	NS
SES → Health Coverage	0.150	0.040	3.788	0.000	S
SES → Malnutrition	0.122	0.041	2.943	0.003	S
SES → Maternal Nutrition Knowledge	0.155	0.040	3.890	0.000	S
Wealth Status → Child Eating Behaviour	0.037	0.049	0.770	0.441	NS
Wealth Status → Child Feeding Practices	−0.102	0.065	1.569	0.117	NS
Wealth Status → Food Insecurity	0.212	0.038	5.583	0.000	S
Wealth Status → SES	0.086	0.041	2.112	0.035	S

S (β), standardized coefficient; SD, standard deviation; *p* value < 0.05 = significant; →, refers; S, significant; NS, Not Significant.
